# A Butyrate Metabolism‐Related Gene Signature Predicts Prognosis, Immune Landscape, and Immunotherapy Efficacy in Breast Cancer

**DOI:** 10.1002/cam4.71763

**Published:** 2026-03-26

**Authors:** Xu Wang, Xuefeng Zheng, Zhan Tuo, Wenjie Sun, Yexiong Li, Hong Ge, Nannan Zhang

**Affiliations:** ^1^ Department of Radiation Oncology The Affiliated Cancer Hospital of Zhengzhou University & Henan Cancer Hospital Zhengzhou China

**Keywords:** breast cancer, butyrate metabolism, prognostic signature, risk score, tumor immune microenvironment

## Abstract

Emerging evidence highlights the critical role of metabolic pathways in breast cancer (BC) progression. Here, we developed a butyrate metabolism‐specific gene (BMRG) signature to predict clinical outcomes and immunotherapy responses in BC, providing a novel pathway‐focused prognostic tool. Using data from The Cancer Genome Atlas (TCGA) and Gene Expression Omnibus (GEO), we identified 102 butyrate metabolism‐related differentially expressed genes (DEGs) through the intersection of DEGs, WGCNA‐derived key module genes, and BMRGs. Univariate Cox followed by least absolute shrinkage and selection operator (LASSO) analysis identified nine genes to construct a prognostic signature, which served as an independent prognostic factor. Risk stratification revealed distinct immune microenvironment and mutation landscapes between subgroups, with risk scores strongly correlating with immune checkpoint expression. The signature exhibited robust prognostic performance, with AUC values for 3‐, 5‐, and 7‐year overall survival ranging from 0.65–0.69 in TCGA and 0.57–0.77 in independent GEO cohorts. Protein–protein interaction analysis identified ACSL1 as a key hub gene, and functional validation confirmed that ACSL1 knockdown suppressed BC cell proliferation and migration. Our findings establish this novel nine‐gene butyrate metabolism‐specific signature as a promising prognostic biomarker and potential therapeutic target for BC, providing a metabolism‐focused perspective for personalized BC management.

**Abbreviations**
AUC: Area under the curve
BC: Breast cancer
BMR‐DEGs: Butyrate metabolism‐related differentially expressed genes
BMRG: Butyrate metabolism‐related gene
CNVs: Copy number variations
GEO: Gene Expression Omnibus
GSEA: Gene set enrichment analysis
HPA: Human protein atlas
ICB: Immune checkpoint blockade
IPS: Immunophenoscore
LASSO: Least absolute shrinkage and selection operator
OS: Overall survival
PCA: Principal component analysis
PPI: Protein–protein interaction
ROC: Receiver operating characteristic
SCFA: Short‐chain fatty acid
TAMs: Tumor associated macrophages
TCGA: The Cancer Genome Atlas
TIDE: Tumor immune dysfunction and exclusion
TIME: Tumor immune microenvironment
TMB: Tumor mutational burden
WGCNA: Weighted gene coexpression network analysis

## 1 Introduction

Breast cancer (BC) remains the most frequently diagnosed malignancy among women globally and continues to pose a major public health burden [1]. Although advances in systemic therapies have significantly improved survival outcomes [2], recurrence and metastasis driven by therapeutic resistance continue to pose significant challenges [3]. Therefore, identifying robust prognostic biomarkers and uncovering novel therapeutic targets are essential for improving personalized management strategies in BC.

Emerging evidence suggests a strong connection between the human gut microbiota and cancer, largely through microbial metabolites [4]. These metabolites can influence BC risk via immune modulation, host metabolism, and estrogen recycling [5]. Butyrate, a short‐chain fatty acid produced by bacterial fermentation, plays a crucial role in regulating cellular metabolism, epigenetic modification, and immune responses across multiple cancer types [6]. In BC, butyrate exerts its effects through multiple mechanisms, including apoptosis induction, reactive oxygen species (ROS) production, and mitochondrial dysfunction [7, 8]. Butyrate also interacts with G‐protein‐coupled receptors such as GPR41/GPR43 and modulates key oncogenic pathways, including DNA methylation and microRNA regulation [9, 10]. In addition, butyrate has demonstrated synergistic effects with established therapeutic agents, including trastuzumab, retinoids, and histone deacetylase inhibitors [11, 12]. Despite these findings, the prognostic value of butyrate metabolism‐related genes (BMRGs) in BC has not been systematically evaluated, nor has their role in shaping the tumor immune microenvironment (TIME) and influencing immunotherapy response been fully elucidated.

The TIME plays a critical role in BC progression [13], and immune checkpoint blockade (ICB) targeting PD‐1/PD‐L1 has become an important treatment strategy [14]. Aberrant metabolism is increasingly recognized as integral to cancer immune dysfunction [15, 16]; however, the relationship between butyrate metabolism and immune modulation in BC, particularly its impact on the TIME, has not been fully explored.

Although butyrate has recognized biological effects in BC, there has been no comprehensive investigation integrating BMRG into a prognostic framework while simultaneously exploring their association with immune microenvironment modulation and immunotherapy response. In this study, we established a prognostic model based on BMRG derived from univariate and least absolute shrinkage and selection operator (LASSO) Cox regression analyses in the TCGA‐BC cohort and validated in independent GEO datasets. A nine‐gene signature was used to calculate a risk score, and a nomogram incorporating clinical features was developed to enhance prognostic prediction. We further evaluated immune infiltration characteristics, mutation patterns, and immunotherapy responses between high‐ and low‐risk groups. The hub gene ACSL1 was selected for in vitro validation. Our study aims to provide a metabolism‐focused prognostic tool and to offer mechanistic insights into how butyrate metabolism may influence the immune landscape and therapeutic responsiveness in BC, thereby contributing to more precise and individualized treatment strategies.

## 2 Materials and Methods

### 2.1 Data Acquisition

**FIGURE 1:**
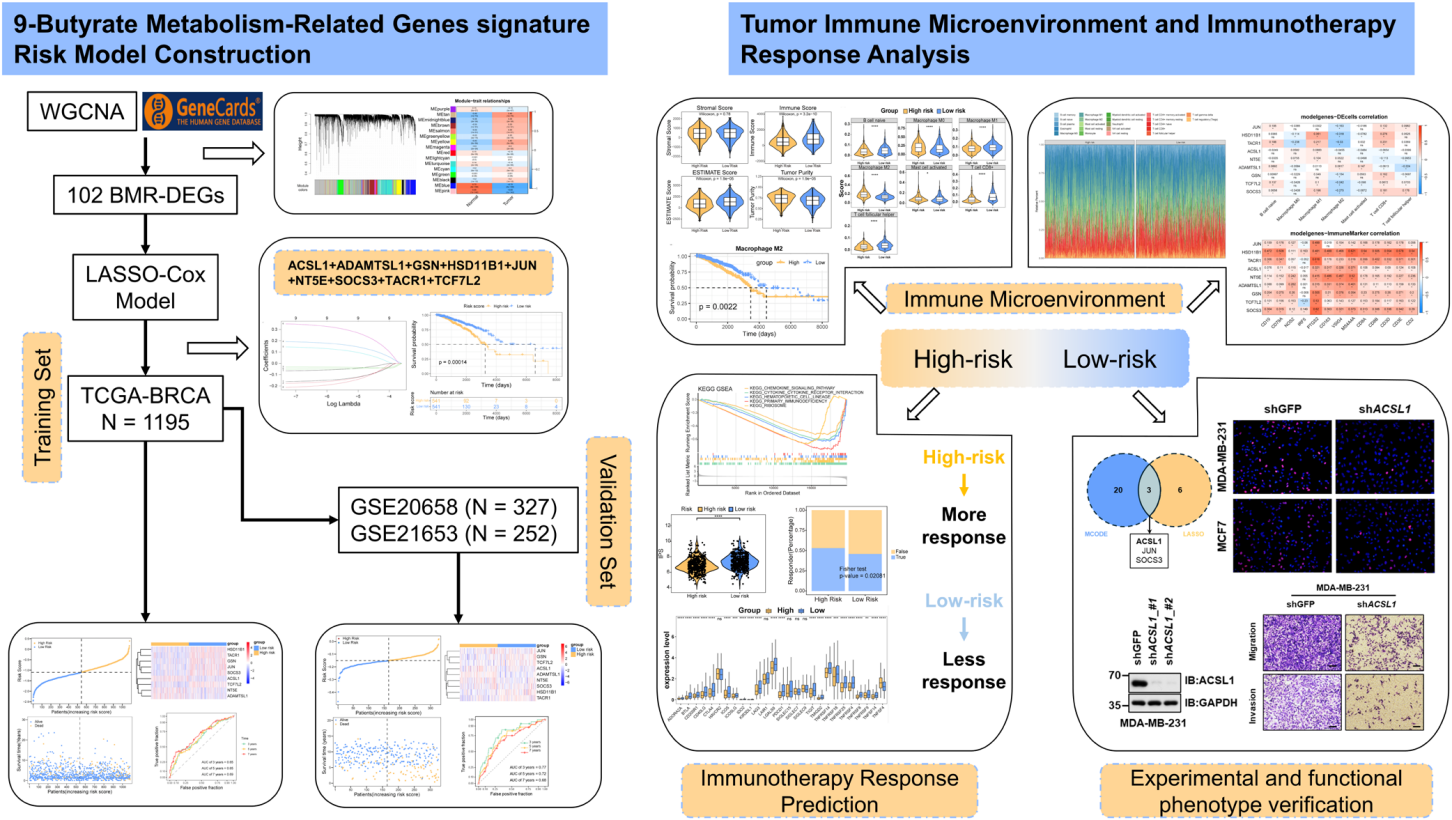
Study design flowchart.

RNA‐sequencing data and corresponding clinical information for BC were obtained from The Cancer Genome Atlas (TCGA), comprising 1082 tumor samples and 113 adjacent normal tissues. Validation datasets GSE20685 (*n* = 327) and GSE21653 (*n* = 252 after filtering) were retrieved from the Gene Expression Omnibus (GEO). GSE70947 (148 tumor and 148 normal samples) was used for expression validation. A total of 882 BMRGs (correlation score > 5) were collected from the GeneCards database. The overall study design is summarized in Figure 1.

The GSE20685 and GSE21653 datasets were specifically selected as independent external validation cohorts due to their matched BC [17, 18]. The sample sizes meet the statistical requirements for the external validation of prognostic models, and both datasets contain gene expression profile data of enrolled patients along with corresponding complete clinical follow‐up information. These cohorts have been widely used in BC prognostic model validation studies, further supporting their suitability for our analysis.

### 2.2 Differential Analysis

Differentially expressed genes (DEGs) between tumor and normal samples from the TCGA cohort were identified using the “DESeq2” R package (v1.36.0) [19], with thresholds of |log2FC| > 1 and adjusted *p*‐value < 0.05. Functional enrichment analysis of DEGs for Gene Ontology (GO) and Kyoto Encyclopedia of Genes and Genomes (KEGG) pathways was performed using “clusterProfiler” (v4.0.5) [20], with an adjusted *p*‐value < 0.05 considered significant.

### 2.3 Weighted Gene Coexpression Network Analysis (WGCNA)

The “WGCNA” package (v1.70‐3) was applied to identify gene modules associated with BC. After sample clustering and outlier removal, a soft‐thresholding power was selected to construct a signed coexpression network. Genes were hierarchically clustered into modules, and the module most significantly correlated with BC was selected for further investigation.

### 2.4 Protein–Protein Interaction (PPI) Network

Butyrate metabolism‐related differentially expressed genes (BMR‐DEGs) were identified by intersecting DEGs, key module genes from WGCNA, and BMRGs. A PPI network was constructed using the STRING database (confidence score > 0.4) and visualized in Cytoscape (v3.10.3). Hub genes were identified using the MCODE plugin.

### 2.5 Risk Score‐Based Subgroup Analysis of BC Patients

A prognostic signature was developed using univariate Cox followed by LASSO regression analyses in the TCGA‐BC cohort. Risk scores for each patient were calculated based on the expression levels and corresponding regression coefficients of the selected genes. Patients were stratified into high‐ and low‐risk groups according to the median risk score within each cohort. In the TCGA‐BC cohort, the median risk score of the training set was used as the cutoff for downstream analyses. For external validation cohorts (GSE20685 and GSE21653), risk stratification was performed using the respective cohort‐specific median risk scores to account for platform heterogeneity. Subsequent immune infiltration and mechanistic analyses (including ESTIMATE and CIBERSORT) were conducted based on the risk grouping defined in the TCGA training cohort, whereas analyses involving IPS and TMB/CNV were performed using cohort‐specific median cutoffs to ensure internal consistency within each dataset. Model performance was evaluated using Kaplan–Meier survival analysis, time‐dependent ROC curves, and further validated in external GEO datasets (GSE20685 and GSE21653). Partial least squares‐discriminant analysis (PLS‐DA) and t‐distributed stochastic neighbor embedding (t‐SNE) were applied to visualize the separation between risk groups.

### 2.6 Mutation Analysis and Nomogram Model Development

Somatic mutations and copy number variations (CNVs) were analyzed using the “maftools” package (v2.10.5) and GISTIC algorithm, respectively. Tumor mutational burden (TMB) was compared between risk groups. Independent prognostic factors identified by univariate and multivariate Cox analyses were incorporated into a nomogram to predict 3‐, 5‐, and 7‐year overall survival (OS). Calibration and ROC curves assessed predictive accuracy.

### 2.7 Gene Set Enrichment Analysis (GSEA)

GSEA was performed using the “clusterProfiler” package to identify signaling pathways differentially activated between risk subgroups, with the KEGG gene set (c2.cp.kegg.v7.5.1.symbols.gmt) as reference and an adjusted *p*‐value < 0.05 considered significant.

### 2.8 Immune Feature Estimation Analysis

Immune infiltration profiles were generated using the CIBERSORT algorithm (implemented via immunedeconv package v2.0.4) to quantify 22 immune cell types, and the ESTIMATE algorithm (estimate package v1.0) was applied to calculate stromal score, immune score, ESTIMATE score, and tumor purity. Correlations between prognostic genes and differentially infiltrated immune cells were assessed, along with their established biomarkers [21]. The expression of 27 immune checkpoints was compared between subgroups [22]. Immunophenoscore (IPS), ESTIMATE score, immune score, and tumor purity were further evaluated.

### 2.9 Analysis Based on Human Protein Atlas (HPA) Database

Protein expression patterns and subcellular localization of the nine prognostic BMRG were validated using immunohistochemistry images from the HPA database.

### 2.10 Cell Culture, Transfection and Infection

Human BC cell lines (MDA‐MB‐231, BT‐549, MCF‐7) and the normal breast epithelial cell line MCF‐10A were cultured under standard conditions. HEK293T cells were kindly provided by Dr. Jin‐Fang Zhang from Wuhan University. Lentiviral particles carrying ACSL1‐targeting shRNAs (shACSL1_#1: 5′‐GCCCAGATGATACTTTGATAT‐3′; shACSL1_#2: 5′‐CCTGTGGGATAAACTCATCTT‐3′) were packaged in HEK293T cells and used to infect MDA‐MB‐231 and MCF‐7 cells, followed by puromycin selection.

### 2.11 RNA Isolation and Quantitative Real‐Time Polymerase Chain Reaction (RT‐qPCR)

Total RNA was extracted using TRIzol reagent, and cDNA was synthesized for RT‐qPCR analysis. Gene expression was normalized to GAPDH and calculated using the 2^−ΔΔ*Ct*^ method. The primers used in this study are listed in Table S1.

### 2.12 Western Blotting

For western blotting, proteins were separated by SDS‐PAGE, transferred to PVDF membranes, and probed with antibodies against ACSL1 and GAPDH. The following antibodies were used: ACSL1 (A16253, 1:1000) and GAPDH (A19056, 1:100,000). All uncropped Western blot images are available in the Supporting Information.

### 2.13 Functional Assays

Cell proliferation was assessed by CCK‐8 and colony formation assays. EdU incorporation was used to evaluate DNA synthesis. Migration capacity was determined by wound healing assay, while Transwell chambers with or without Matrigel coating were employed to analyze migration and invasion capabilities, respectively. All original microscopic images are available in the Supporting Information.

### 2.14 Statistical Analysis

All statistical analyses were performed using R software (v4.2.1) and GraphPad Prism 7.0. For bioinformatic analyses (including differential expression, WGCNA, risk model construction, immune infiltration, and CNV/TMB analysis), data preprocessing involved format conversion, log2 transformation (where applicable), and normalization, with outlier assessment limited to sample/gene missing value removal. Data were visualized via heatmaps, volcano plots, survival curves, and violin/box plots, with descriptive statistics (mean ± SD) reported only for in vitro experiments. Sample sizes varied by analysis: TCGA cohort included 1082 tumor and 113 normal samples, GEO validation cohorts (GSE20685/GSE21653) had 327/252 samples, and in vitro experiments used *n* = 3 independent replicates. Statistical methods included two‐sided tests (Wald test for differential expression, Cox proportional hazards regression for survival, Wilcoxon rank‐sum test for group comparisons, Fisher's exact test for categorical data) with Benjamin–Hochberg/FDR correction for multiple comparisons; assumptions for parametric tests were assumed to be met. Key R packages included DESeq2 (v1.36.0), WGCNA (v1.70‐3), clusterProfiler (v4.0.5), survival (v3.2‐13), survminer (v0.4.9), maftools (v2.10.5), while GraphPad Prism was used for experimental data analysis.

## 3 Results

### 3.1 Identification of BMR‐DEGs in the TCGA‐BC Dataset

**FIGURE 2:**
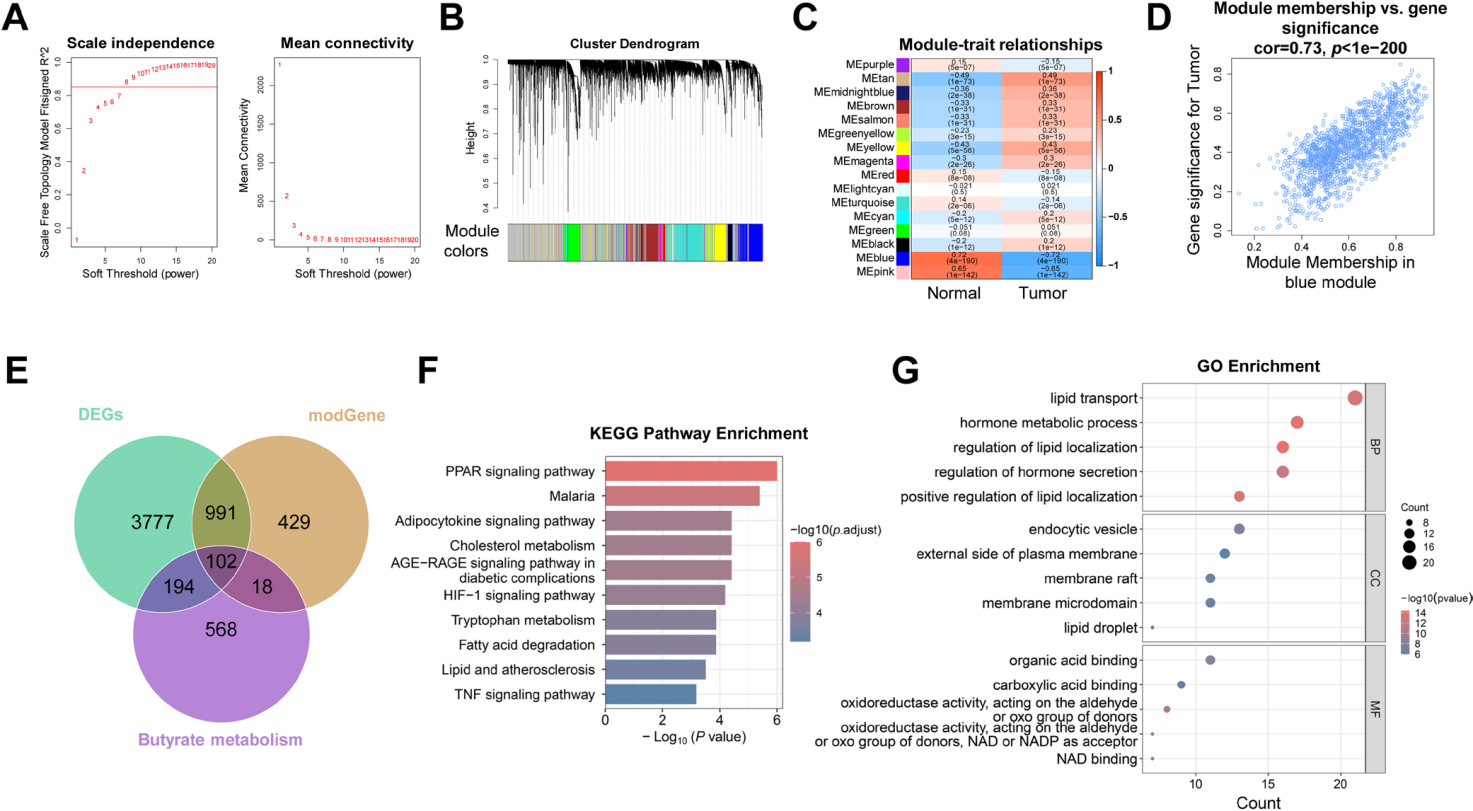
Identification of candidate BMR‐DEGs in TCGA‐BC cohort. (A) Determination of soft‐threshold power in WCGNA. In all, 8 was the most fit power value. (B) Gene clustering dendrogram and module assignment. (C) Module‐trait relationships identifying the blue module as most correlated with BC. (D) Correlation between gene significance and module membership in the blue module. (E) Venn diagram identifying BMR‐DEGs. (F, G) Enrichment analysis using the KEGG and GO database.

A total of 5064 DEGs were identified from the TCGA‐BC dataset, comprising 3052 upregulated and 2012 downregulated genes (Figure S1A). Top 20 genes (top 10 upregulated and top 10 downregulated) were shown by heat map (Figure S1B). Then we did a WGCNA to identify key BC modules. After confirming no outlier samples (Figure S1C), a soft threshold power of 8 was selected based on scale‐free topology criterion (signed *R* ^2^ = 0.85) (Figure 2A). Dynamic tree cutting identified 16 coexpression modules (Figure 2B), among which the MEblue module demonstrated the strongest correlation with BC (correlation coefficient = 0.73, *p* < 0.0001, Figure 2C,D). This yielded 1540 key module genes for subsequent analysis (Figure 2D). Intersection of these module genes with DEGs and BMRGs identified 102 butyrate metabolism‐related DEGs (BMR‐DEGs) (Figure 2E). Functional enrichment analysis revealed these genes were significantly enriched in lipid metabolism pathways including “cholesterol metabolism,” “fatty acid degradation,” and “lipid and atherosclerosis,” as well as key signaling pathways such as “PPAR signaling,” “HIF‐1 signaling,” and “TNF signaling” (Figure 2F). GO analysis indicated predominant involvement in lipid transport and hormone metabolic processes (Figure 2G), suggesting crucial roles in lipid metabolism regulation and signal transduction.

### 3.2 Construction and Evaluation of a Prognostic BMRG Signature Based on BMR‐DEGs

**FIGURE 3:**
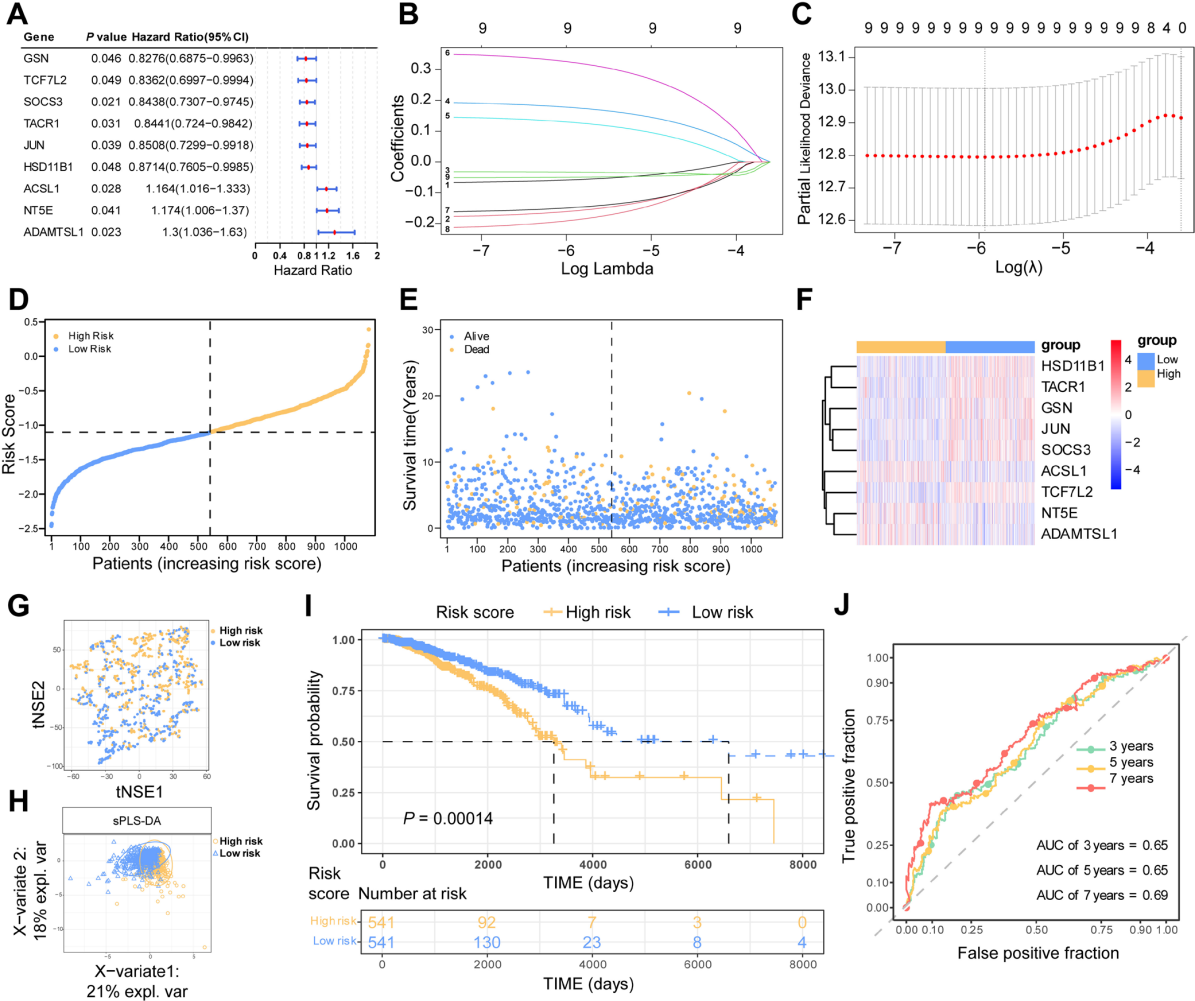
Construction of BMRG prognostic signature. (A) Univariate Cox regression analysis of BMR‐DEGs (*n* = 1082). (B) LASSO coefficient profiles of the nine BMRG. (C) Cross‐validation for tuning parameter selection. (D) Risk score distribution and cutoff (high‐risk, *n* = 541; low‐risk, *n* = 541). (E) Survival status and risk score distribution. (F) Expression heatmap of the nine prognostic BMRG. (G) t‐NSE analysis plot. (H) PCA analysis plot. (I) Kaplan–Meier survival analysis. (J) Time‐dependent ROC curves.

Univariate Cox analysis of the 102 BMR‐DEGs identified nine genes significantly associated with prognosis in the TCGA‐BC cohort (Figure 3A). And we identified ACSL1, NT5E, and ADAMTSL as significant risk factors in our analysis. Subsequent LASSO regression analysis confirmed these nine genes—ACSL1, ADAMTSL1, GSN, HSD11B1, JUN, NT5E, SOCS3, TACR1, and TCF7L2—as the optimal feature set for constructing the BMRG signature (Figure 3B,C). The risk score for each patient was calculated as the weighted sum of the expression levels of the nine prognostic BMRG, with the specific regression coefficients for each gene listed in Table S2, and patients were stratified into high‐ and low‐risk groups based on the median score (*n* = 541 each; Figure 3D). The distribution of survival status across risk groups is shown in Figure 3E, with high expression of HSD11B1, TACR1, GSN, JUN, SOCS3, and TCF7L2 predominantly observed in the low‐risk group (Figure 3F). Both t‐SNE and PCA demonstrated clear separation between the two risk groups (Figure 3G,H). Kaplan–Meier analysis revealed significantly poorer OS in the high‐risk group (Figure 3I). Time‐dependent ROC curves confirmed the model's predictive accuracy for 3‐, 5‐, and 7‐year survival, with AUC values of 0.65, 0.65, and 0.69, respectively (Figure 3J), supporting the reliability of the BMRG signature in prognostic stratification.

### 3.3 Validation of the Prognostic Model in the GEO Cohort

**FIGURE 4:**
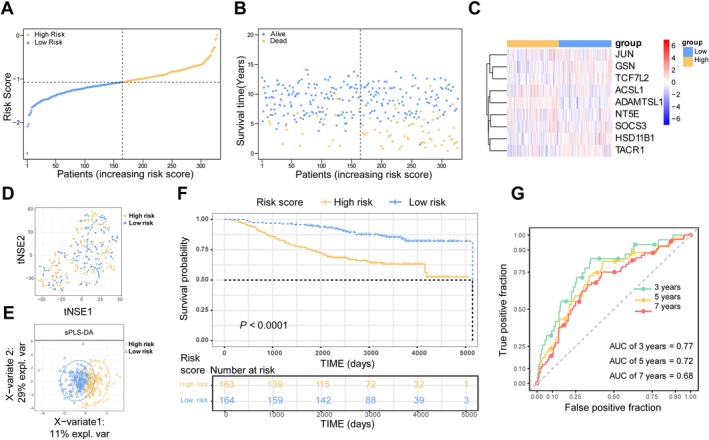
Validation of the BMRG signature in GEO cohort. (A) Risk score distribution (High‐risk, *n* = 163; Low‐risk, *n* = 164). (B) Survival status and risk score distribution. (C) BMRG expression heatmap. (D) t‐NSE analysis plot. (E) PCA analysis plot. (F) Kaplan–Meier survival analysis. (G) Time‐dependent ROC curves.

The prognostic model was further validated in two independent GEO datasets (GSE20685 and GSE21653). In GSE20685, patients were stratified into high‐risk (*n* = 163) and low‐risk (*n* = 164) groups based on the median risk score (Figure 4A). The distribution of survival status and risk scores is shown in Figure 4B, while expression patterns of the nine BMRG were consistent with the TCGA cohort (Figure 4C). Clear separation between risk groups was observed in t‐SNE and PCA plots (Figure 4D,E). Patients in the high‐risk group showed significantly poorer OS (Figure 4F), with the model demonstrating strong predictive accuracy (3‐, 5‐, and 7‐year AUCs: 0.77, 0.72, and 0.68, respectively; Figure 4G).

Similar results were observed in GSE21653, where high‐risk patients (*n* = 126) also exhibited significantly worse survival outcomes compared to low‐risk patients (*n* = 126) (Figure S2A–F). The model maintained acceptable predictive performance in this cohort, with 3‐, 5‐, and 7‐year AUCs of 0.57, 0.59, and 0.62, respectively (Figure S2G). These consistent findings across independent datasets confirm the robustness and generalizability of the BMRG‐based prognostic signature.

### 3.4 Mutation Landscape Analysis Between Two Risk Subgroups in BC

**FIGURE 5:**
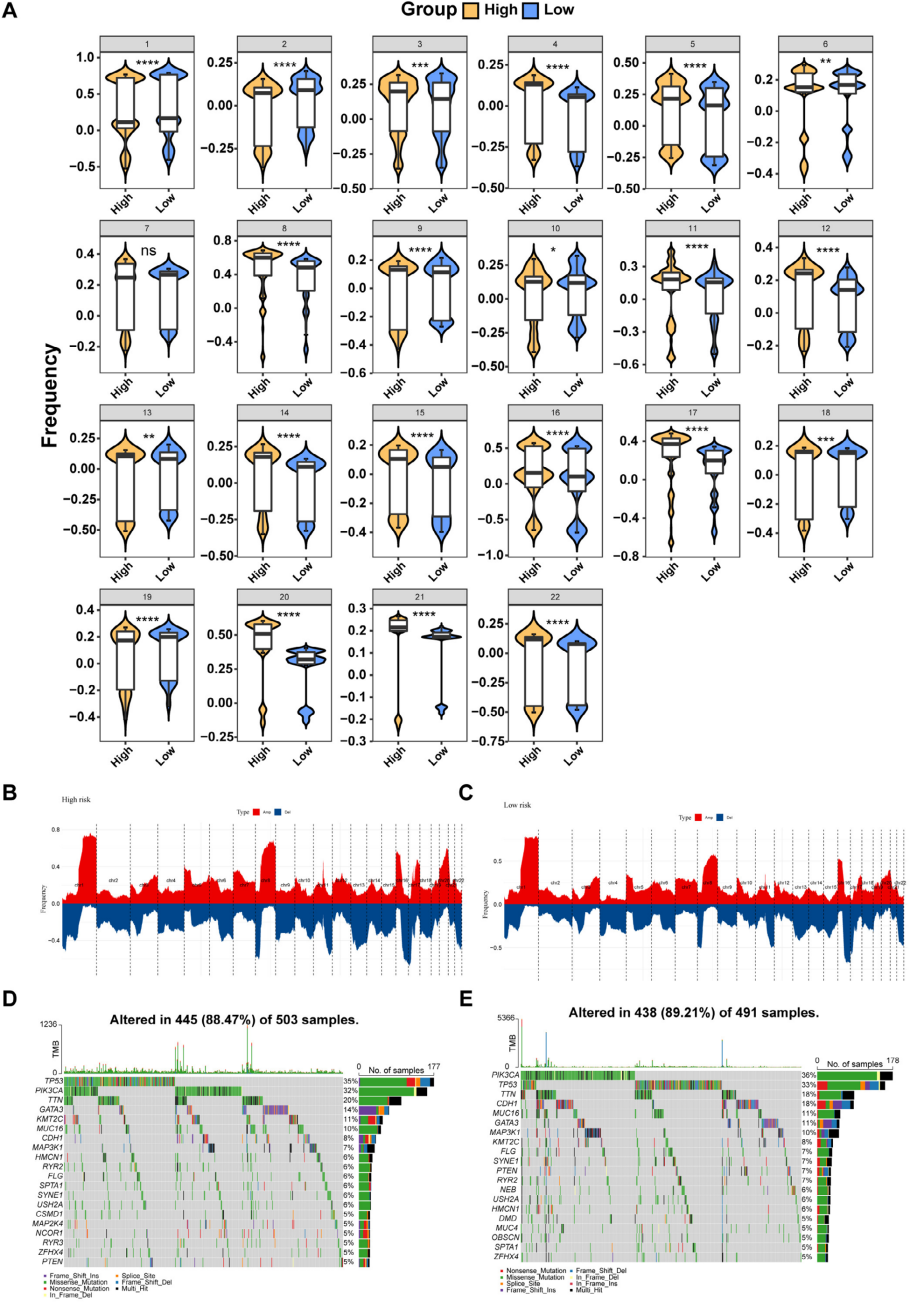
Mutation landscape comparison between risk subgroups. (A) Copy number variation (CNV) frequency between high‐ and low‐risk groups (Data are presented as mean ± SD. Statistical significance was determined by Wilcoxon rank‐sum test, with *p < 0.05, **p < 0.01, ***p < 0.001, ****p < 0.0001). (B) Chromosomal CNV profile in high‐risk group. (C) Chromosomal CNV profile in low‐risk group. (D) Somatic mutation landscape in high‐risk group. (E) Somatic mutation landscape in low‐risk group.

We analyzed the genomic mutation landscape to compare molecular features between risk subgroups. The high‐risk group exhibited more extensive CNVs across all autosomes except chromosome 7 (Figure 5A–C). However, the overall mutation rates were comparable between subgroups (88.47% in high‐risk vs. 89.21% in low‐risk, Figure 5D,E), suggesting that prognostic differences are not attributable to mutation burden.

### 3.5 Independent Prognostic Analysis for BC Patients and Creation of the BMGRs‐Based Nomogram

**FIGURE 6:**
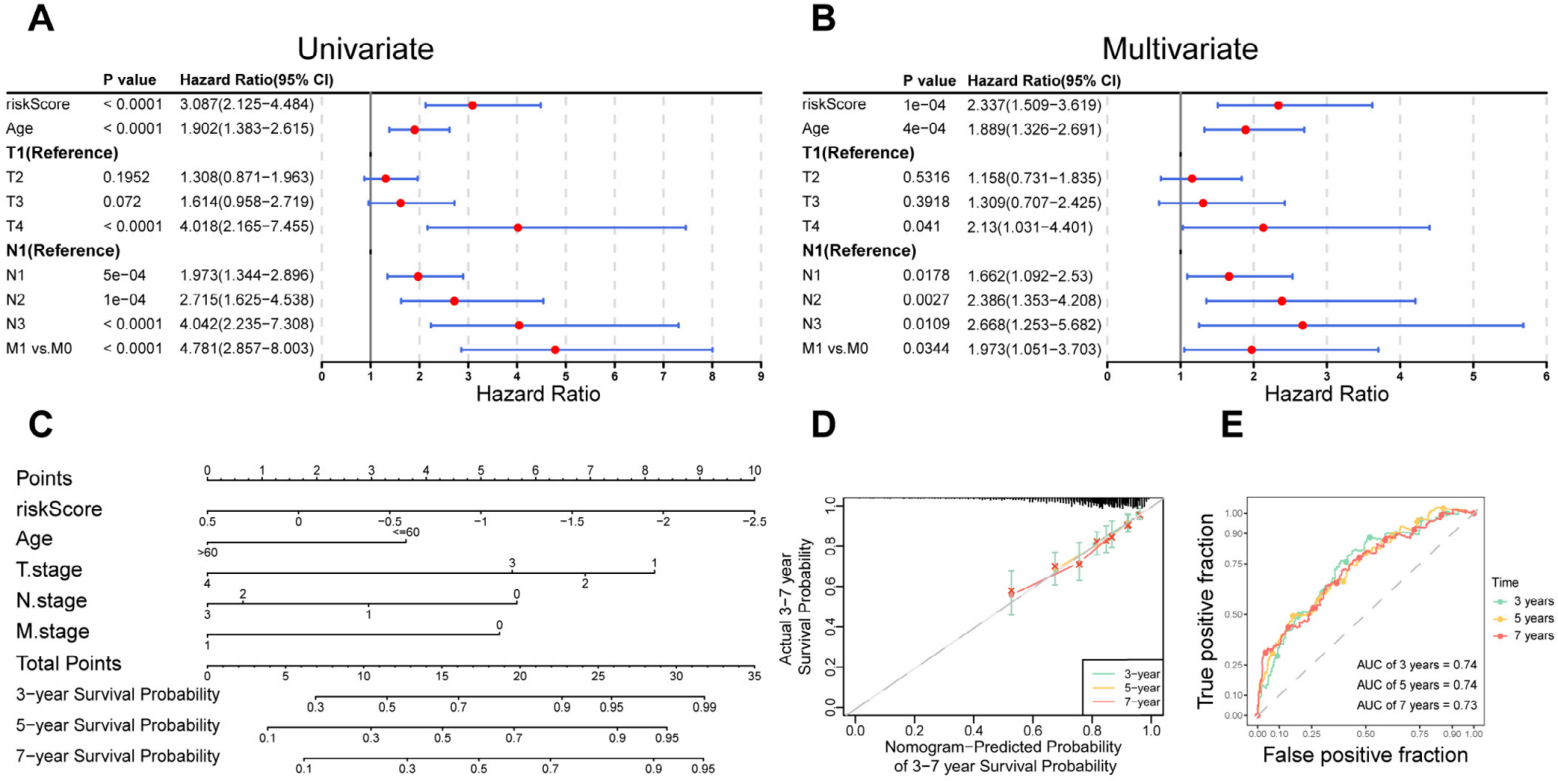
Independent prognostic role of risk scores. (A) Univariate Cox regression analysis. (B) Multivariate Cox regression analysis. (C) Prognostic nomogram. (D) Calibration curves. (E) ROC analysis.

Univariate and multivariate Cox analyses identified age, risk score, and pathologic T/N/M stages as independent prognostic factors in the TCGA‐BC cohort (Figure 6A,B). These variables were incorporated into a BMRG‐based nomogram to predict 3‐, 5‐, and 7‐year OS probabilities (Figure 6C). The calibration and ROC curves demonstrated that the nomogram was reliable (Figure 6D,E). Consistent results were obtained in the GSE20685 validation cohort, where risk score and pathologic T/N/M stages remained independently prognostic (Figure S3A–B), and the corresponding nomogram also exhibited reliable performance (Figure S3C–E).

### 3.6 Correlation Between BMRG Signature and Immune Infiltration in BC

**FIGURE 7:**
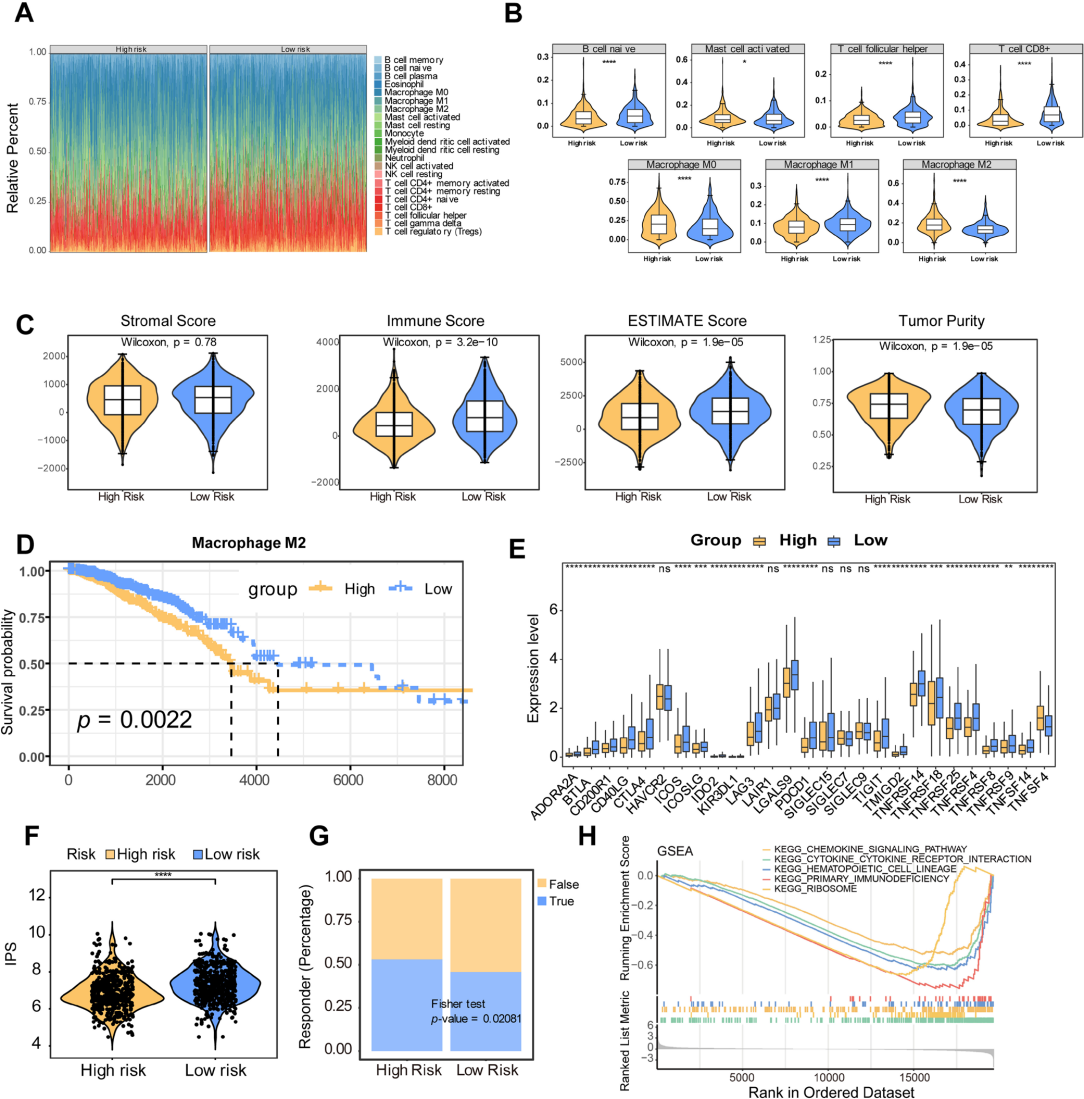
Association between BMRG signature and tumor immune microenvironment. (A) Immune cell infiltration patterns (high‐risk, *n* = 541; low‐risk, *n* = 541). (B) Differentially infiltrated immune cells. (C) Tumor microenvironment scores. (D) Survival analysis based on M2 macrophage infiltration (Statistical significance was determined by log‐rank test, with *p* < 0.05). (E) Immune checkpoint expression. (F) Immunophenoscore comparison. (G) Immunotherapy response prediction. (H) GSEA pathway enrichment (B, C, E and F are presented as mean ± SD. Statistical significance was determined by Wilcoxon rank‐sum test, with *p < 0.05, **p < 0.01, ***p < 0.001, ****p < 0.0001).

Analysis of the TIME revealed significant differences in immune cell composition between risk groups. Seven immune cell types showed distinct infiltration patterns, including naive B cells, macrophage subtypes (M0, M1, M2), CD8^+^ T cells, activated mast cells, and follicular helper T cells (Figure 7A,B). The high‐risk group exhibited significantly lower immune and ESTIMATE scores but higher tumor purity (Figure 7C). Correlation analysis identified significant associations between prognostic genes and immune features. HSD11B1 expression positively correlated with M1 macrophages and CD8^+^ T cells, while TACR1 and HSD11B1 showed negative correlations with M2 macrophages (Figure S4A). Consistent with these findings, HSD11B1 strongly correlated with CD8^+^ T‐cell markers CD8A and CD8B (Figure S4B). Survival analysis indicated that M2 macrophage infiltration was associated with poor prognosis (Figure 7D).

Notably, 22 immune checkpoints including CTLA4, LAG3, PDCD1 (PD‐1), and TIGIT were differentially expressed between risk groups, with lower IPS in high‐risk patients (Figure 7E,F). However, TIDE analysis interestingly suggested better response to ICB in the high‐risk group, reflecting differences in the assessment frameworks of the two predictive tools (Figure 7G). GSEA revealed enhanced chemokine and cytokine signaling in low‐risk patients, while cell cycle pathways were enriched in high‐risk patients (Figure 7H and Figure S4F). Validation in GSE20685 confirmed differential expression of eight immune checkpoints and identified memory B cells and M2 macrophages as key distinguishing immune subsets (Figure S4C–E).

### 3.7 Validation of the Prognostic Genes in the Clinical Samples and RT‐qPCR

**FIGURE 8:**
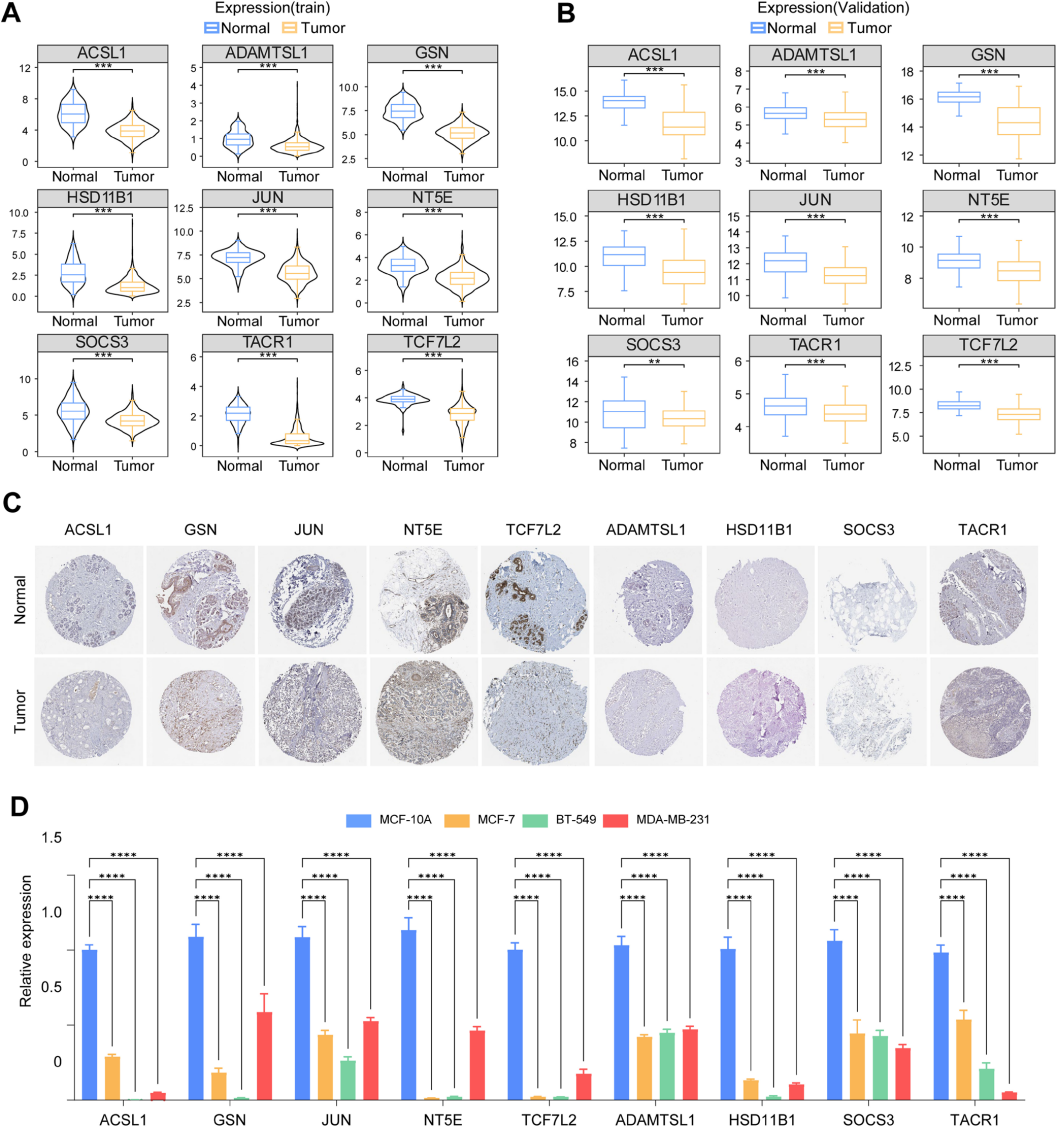
Validation of BMRGs expression patterns. (A) BMRG expression in TCGA cohort (normal, *n* = 113; tumor, *n* = 1082). (B) BMRG expression in GEO cohort (normal, *n* = 148; tumor, *n* = 148). (C) Protein expression validation using HPA database. (D) mRNA expression validation by RT‐qPCR (data are presented as mean ± SD. Statistical significance was determined by *t* test, with *p < 0.05; **p < 0.01; ***p < 0.001; ****p < 0.0001).

Expression of the nine prognostic BMRG was consistently downregulated in BC tissues compared to normal controls across both training and validation datasets at the transcriptional level (Figure 8A,B). Immunohistochemistry results from the HPA database further confirmed markedly reduced protein expression of ACSL1, GSN, JUN, NT5E, and TCF7L2 in BC specimens, while ADAMTSL1, HSD11B1, SOCS3, and TACR1 showed less pronounced differences (Figure 8C). RT‐qPCR analysis in multiple BC cell lines validated the downregulated mRNA expression pattern of all nine BMRG, aligning with the bioinformatic predictions (Figure 8D). Collectively, these results reinforce the stability and reliability of the BMRG signature.

### 3.8 Drug Sensitivity Analysis of Hub Gene ACSL1 and Predicted a Worse Prognosis for BC Patients

**FIGURE 9:**
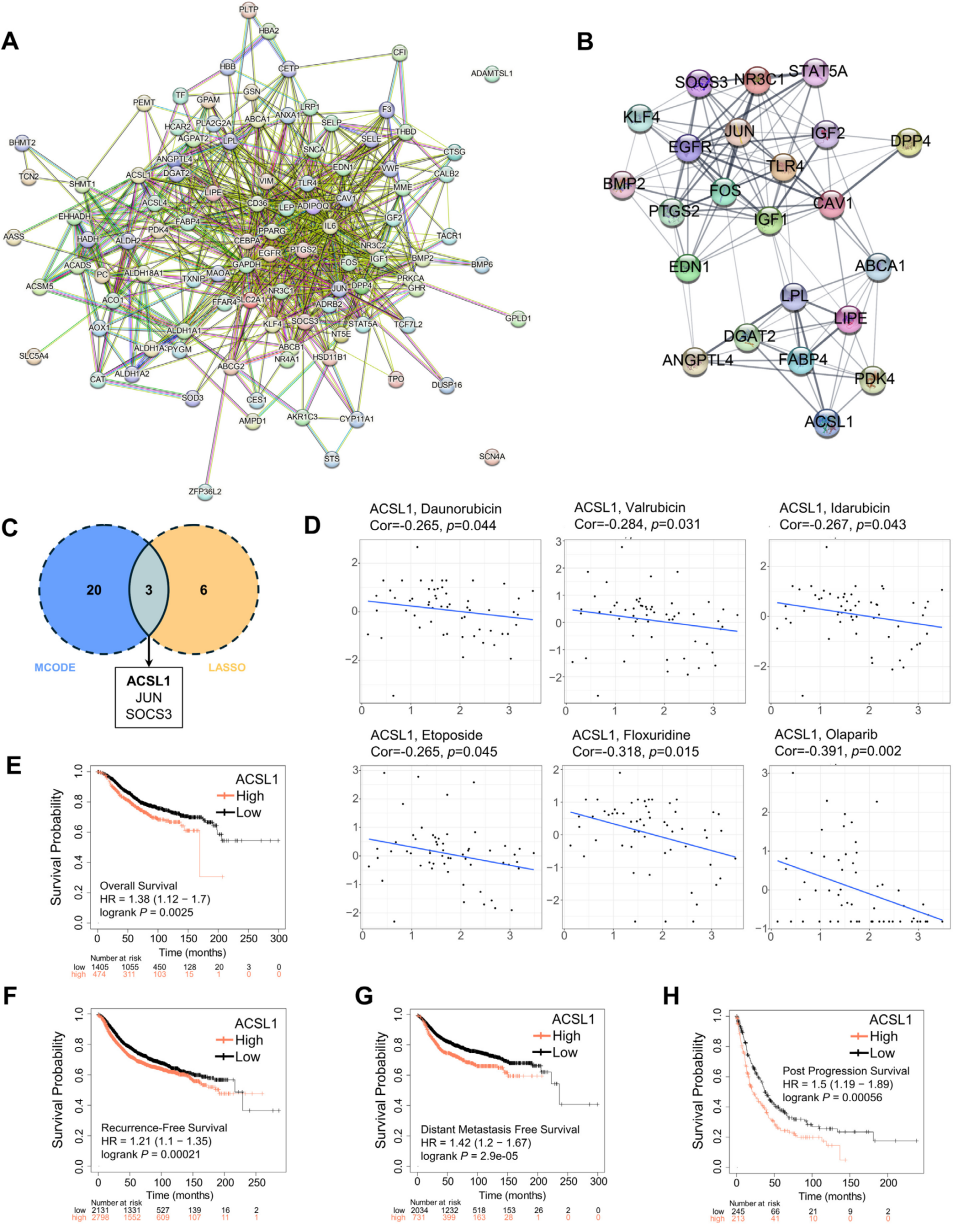
Clinical and therapeutic significance of ACSL1. (A) The PPI network of BMR‐DEGs. (B) Key functional module identification. (C) Venn diagram showed core genes of BMRG. (D) Drug sensitivity of ACSL1 in the Cell Miner database. (E–H) Survival analysis based on ACSL1 expression (Statistical significance was determined by log‐rank test, with *p* < 0.05).

To investigate the regulatory interactions, a PPI network of the BMR‐DEGs was constructed, consisting of 102 nodes and 643 edges (Figure 9A). The correlation patterns of BMR‐DEGs were further visualized in Figure S4G. Utilizing the MCODE plugin, key functional modules were identified, with the most significant module containing 23 nodes and 111 edges (Figure 9B). An intersection analysis between these 23 MCODE‐derived genes and the BMRG selected via LASSO regression revealed three core genes: ACSL1, JUN, and SOCS3 (Figure 9C). Our previous findings revealed that ACSL1 exhibited a prominent positive risk coefficient in the prognostic signature, and univariate Cox regression analysis confirmed ACSL1 as a significant independent prognostic factor for BC patients. Subsequent studies identified ACSL1 as a key hub gene in the PPI network of BMR‐DEGs, and our subsequent functional analyses thus focused on ACSL1. Using the CellMiner database, we examined the relationship between ACSL1 expression and drug sensitivity. Notably, ACSL1 expression showed a significant negative correlation with sensitivity to multiple chemotherapeutic agents, including daunorubicin, valrubicin, idarubicin, etoposide, floxuridine, and olaparib (Figure 9D), suggesting that ACSL1 may contribute to chemotherapy resistance in BC patients.

Furthermore, we utilized the Kaplan–Meier plotter database to assess the impact of ACSL1 on the survival of BC patients [23]. The KM curves revealed that patients with high ACSL1 expression had significantly shorter OS, recurrence‐free survival (RFS), distant metastasis‐free survival (DMFS), and postprogression survival (PPS) compared to those with low ACSL1 expression (Figure 9E–H). Collectively, these findings highlight ACSL1 as a potential therapeutic target and prognostic marker in BC.

### 3.9 ACSL1 Promoted Proliferation, Migration and Invasion of BC Cells

**FIGURE 10:**
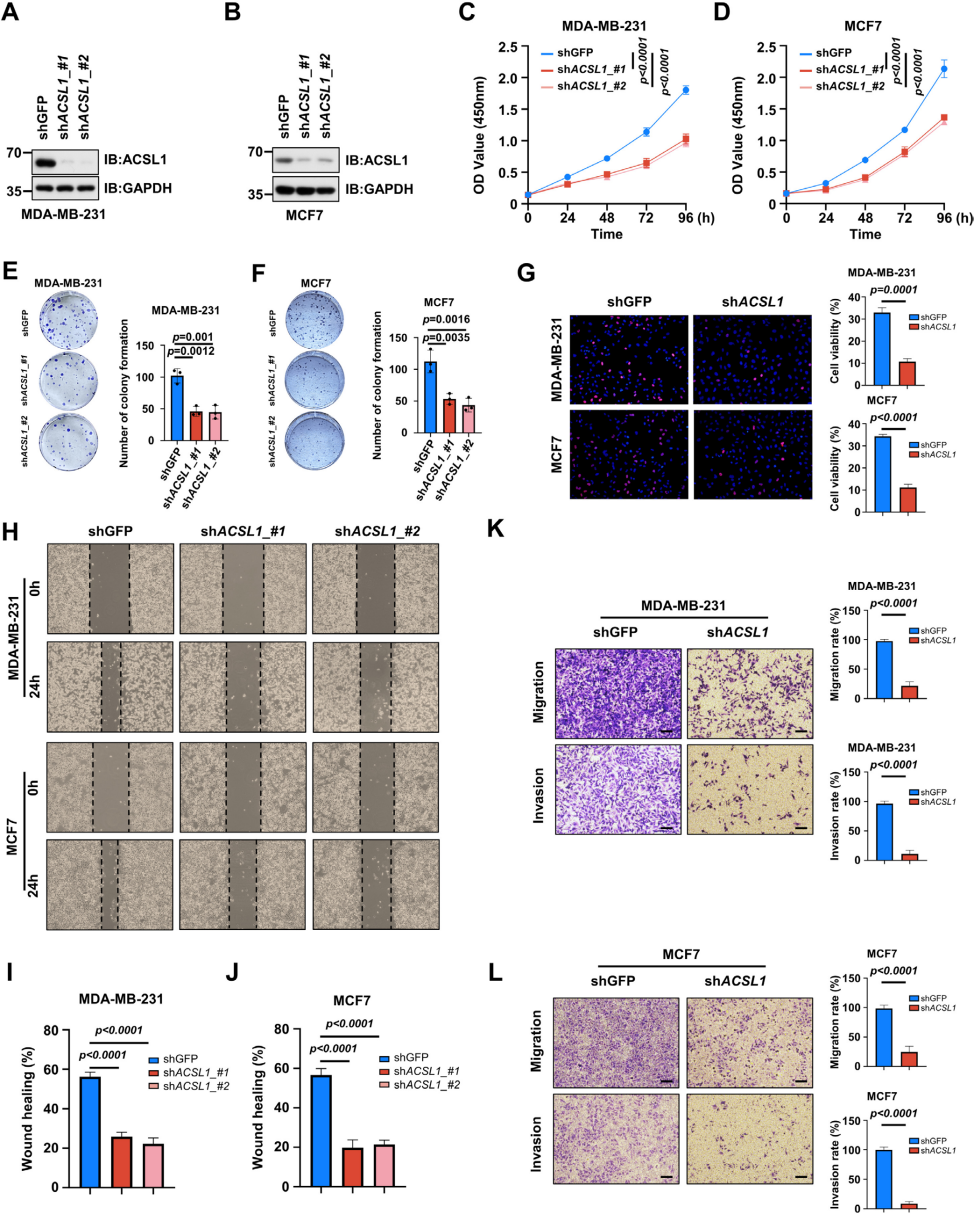
Functional validation of ACSL1 in BC cells. (A, B) ACSL1 knockdown efficiency. (C, D) Cell viability analysis. (E, F) Colony formation assay. (G) EdU proliferation assay. (H–J) Wound healing assay. (K, L) Transwell migration and invasion assays. (All uncropped Western blot images and original microscopic images are available in the Supporting Information. Data are presented as mean ± SD. Statistical significance was determined by two‐tailed Student's *t*‐test with *n* = 3 independent experiments, with *p < 0.05, **p < 0.01, ***p < 0.001, ****p < 0.0001).

To functionally characterize ACSL1 in BC progression, we established stable ACSL1‐knockdown models in MDA‐MB‐231 and MCF‐7 cells, with efficient knockdown confirmed by western blotting (Figure 10A,B). Functional assays demonstrated that ACSL1 depletion significantly suppressed cellular proliferation, as evidenced by CCK‐8 viability assays (Figure 10C,D), colony formation capacity (Figure 10E,F), and EdU incorporation rates (Figure 10G).

Furthermore, ACSL1 knockdown markedly impaired migratory ability in wound healing assays (Figure 10H–J) and reduced both migration and invasion in transwell assays (Figure 10K,L). These consistent findings across multiple cell lines confirm that ACSL1 plays a crucial role in promoting proliferative and metastatic phenotypes in BC cells.

## 4 Discussion

BC remains a leading cause of cancer‐related mortality among women worldwide [24]. Although numerous prognostic biomarkers have been proposed [25], clinically robust tools that integrate tumor metabolism and immune regulation remain limited. In this study, we developed and validated a BMRG signature and demonstrated its prognostic value as well as its close association with the TIME, thereby providing a metabolism‐centered framework for risk stratification in BC.

Rather than functioning as isolated markers, the nine genes included in our signature converge on interconnected metabolic and regulatory pathways. Notably, ACSL1, HSD11B1, and NT5E represent central nodes linking butyrate metabolism to lipid remodeling and energy reprogramming. ACSL1 is a key regulator of long‐chain fatty acid activation and has been implicated in ferroptosis modulation and metabolic plasticity in cancer [26, 27]. HSD11B1 participates in steroid and metabolic homeostasis and has been associated with altered tumor cell metabolic states in BC [28, 29]. NT5E (CD73), beyond its established role in adenosine production and immune suppression, promotes tumor growth through AKT/GSK‐3β/β‐catenin signaling [30]. Collectively, these genes suggest that the prognostic performance of the BMRG signature reflects a coordinated butyrate–lipid metabolism axis that may influence tumor proliferation, metabolic flexibility, and immune modulation. Additional members of the signature, including JUN, TCF7L2, and SOCS3, further connect metabolic signaling with transcriptional regulation, inflammatory responses, and stemness‐related pathways [31, 32, 33, 34]. Thus, the biological relevance of the model likely arises from a coordinated metabolic‐regulatory network rather than the effect of individual genes, reinforcing its mechanistic plausibility.

Emerging evidence indicates that microbial metabolites influence tumor responsiveness to ICB [35]. Our findings extend this concept by demonstrating that BMRGs are closely linked to immune landscape remodeling in BC. High‐risk patients exhibited lower immune and ESTIMATE scores together with higher tumor purity, consistent with an immunologically “cold” phenotype. This group showed increased infiltration of M0/M2 macrophages and activated mast cells, alongside reduced B cells, M1 macrophages, CD8^+^ T cells, and T follicular helper (Tfh) cells. Among these alterations, the predominance of M2‐like tumor‐associated macrophages (TAMs) appears central to the adverse prognostic profile. M2 TAMs suppress cytotoxic T‐cell activity and promote immune evasion [36, 37], and our survival analysis confirmed their association with poorer outcomes. Conversely, the reduced infiltration of Tfh cells in high‐risk tumors may weaken coordinated B‐cell and CD8^+^ T‐cell responses [38], thereby limiting effective antitumor immunity. These findings suggest that dysregulated butyrate metabolism may contribute to the establishment of an immunosuppressive metabolic niche, reinforcing tumor progression through metabolic–immune crosstalk.

Despite the expanding application of ICB therapies in BC [39], predictive biomarkers remain incompletely defined. We observed differential expression of multiple immune checkpoint genes, including PD‐1, LAG3, TIGIT, and CTLA4, with generally higher expression in low‐risk patients, suggesting a relatively immune‐active microenvironment in this subgroup. Interestingly, predictive algorithms yielded partially discordant results: high‐risk patients showed lower IPS yet were predicted by TIDE analysis to have better responsiveness to ICB. This discrepancy highlights the multidimensional nature of immune prediction. IPS primarily reflects tumor immunogenicity and effector cell activity, whereas TIDE emphasizes immune dysfunction and exclusion mechanisms. Therefore, immune checkpoint expression or immunogenicity alone may not fully determine therapeutic efficacy. Instead, the broader metabolic context—particularly the degree of immune suppression shaped by metabolic reprogramming—may play a decisive role in modulating treatment response. Consistently, GSEA demonstrated enrichment of chemokine and cytokine signaling pathways in low‐risk tumors, supporting enhanced immune recruitment [40, 41], whereas high‐risk tumors were enriched in cell cycle‐related pathways, reflecting proliferative dominance over immune activation. Notably, tumor mutation burden did not differ significantly between risk groups, aligning with findings from the IMpassion130 trial [42] and suggesting that TMB may have limited predictive value for ICB response in BC. Together, these observations reinforce the concept that metabolic features may provide complementary predictive information beyond genomic mutation metrics.

Among the signature genes, ACSL1 emerged as a pivotal metabolic regulator with functional relevance. Previous studies in multiple malignancies have demonstrated its role in fatty acid metabolism reprogramming, ferroptosis resistance, and tumor progression [26, 43, 44, 45, 46]. Our results confirm its association with poor prognosis in BC and provide experimental evidence that ACSL1 promotes tumor cell proliferation and epithelial–mesenchymal transition. Importantly, ACSL1 may serve as a metabolic–immune hub: by modulating lipid metabolic flux and inflammatory signaling, it may reshape the tumor microenvironment and sustain a protumorigenic niche. These findings suggest that targeting ACSL1 or related metabolic pathways could represent a promising therapeutic strategy, particularly in metabolically defined high‐risk subgroups.

Taken together, our study does more than propose a prognostic model; it provides evidence that butyrate metabolism reprogramming may contribute to BC progression by orchestrating lipid metabolic remodeling and immune suppression within the tumor microenvironment. This integrated metabolic–immune perspective may facilitate more precise patient stratification and inform the development of personalized immunotherapeutic strategies.

Our study has several limitations. First, all analyses of the nine‐gene BMRG signature were based on retrospective public omics datasets, and only in silico validation was completed in independent GEO cohorts; due to practical constraints in the acquisition of clinical BC specimens and paired clinical follow‐up data, we did not perform experimental validation of the entire prognostic model using actual clinical patient cohorts and specimens, and the experiment validation in this study was only focused on the key hub gene ACSL1. Reliance on bioinformatic predictions and single‐gene functional validation thus warrants systematic in vitro and in vivo experimental validation of the entire nine‐gene model using clinical specimens in future research. Second, this study lacks direct experimental data on butyrate concentrations and comprehensive metabolomics profiling. Third, reliance on bioinformatic predictions warrants further experimental validation in vivo and in vitro, especially the molecular mechanisms of metabolic‐immune crosstalk mediated by BMRG. In addition, the assessment of tumor immune infiltration and immune scoring was primarily based on CIBERSORT and ESTIMATE algorithms, and the single methodological approach may lead to potential biases in the results. Future work will refine the prognostic model in prospective clinical cohorts and incorporate metabolomics assays to directly measure butyrate levels, adopt multiple complementary immune analysis algorithms for cross‐validation of immune infiltration results, aiming to validate the clinical utility of the BMRG signature and clarify the causal role of butyrate metabolism in BC progression and immunotherapy response.

In conclusion, we developed and performed multicohort bioinformatic validation for a novel prognostic model based on nine BMRG, which effectively stratifies BC patients into distinct risk categories. This signature is centered on butyrate‐mediated lipid metabolic reprogramming, closely linked to the remodeling of the TIME, and its predictive value for prognosis and immunotherapy efficacy is based on the core metabolic‐immune crosstalk in BC. Our findings provide a metabolism‐focused perspective for personalized BC management, and targeting the butyrate metabolism pathway, especially the key hub gene ACSL1, may be a promising strategy to combine metabolic therapy and immunotherapy for BC. In addition, to further clarify the predictive value of these complementary immune indices, future studies should integrate real‐world clinical immunotherapy cohort data to validate the association between the BMRG signature, immune landscape features, and actual ICB treatment outcomes.

## Author Contributions

**Xu Wang:** data curation, investigation, software, visualization, writing – original draft, funding acquisition. **Xuefeng Zheng:** formal analysis, investigation, writing – original draft, visualization. **Zhan Tuo:** supervision, writing – review and editing. **Wenjie Sun:** formal analysis, investigation, funding acquisition. **Yexiong Li:** supervision, funding acquisition. **Hong Ge:** supervision, writing – review and editing. **Nannan Zhang:** conceptualization, investigation, funding acquisition, writing – review and editing. Xu Wang and Xuefeng Zheng contributed equally to this work. All authors read and approved the final manuscript.

## Funding

This work was supported by National Science and Technology Major Project of the Ministry of Science and Technology of China (grant numbers: 2023ZD0502200), National Natural Science Foundation of China (grant numbers: 82203285, 82302951) and Key Technologies R & D Program of the Henan Provincial Department of Science and Technology (grant numbers: 222102310350, 222102310432), and Natural Science Foundation of Henan Province (grant numbers 242300421508).

## Ethics Statement

The authors have nothing to report.

## Consent

The authors have nothing to report.

## Conflicts of Interest

The authors declare no conflicts of interest.

## Supporting information


**Figure S1:** Screening of BMR‐DEGs in TCGA‐BC cohort.
**Figure S2:** Validation of BMRG signature in GSE21653 cohort.
**Figure S3:** Nomogram construction in GSE20685 validation set.
**Figure S4:** Immune infiltration and functional analysis.
**Table S1:** Primer sequences used in this study.
**Table S2:** Gene Coefficients and Risk Effects.

## Data Availability

The datasets used and/or analyzed during the current study can be made available from the corresponding author on reasonable request. We obtained the mRNA sequencing data of BC samples from the Gene Expression Omnibus (GEO) database (https://www.ncbi.nlm.nih.gov/geo/).
