# Supplementary material for: A Butyrate Metabolism‐Related Gene Signature Predicts Prognosis, Immune Landscape, and Immunotherapy Efficacy in Breast Cancer
Source: Cancer Med. 2026 Mar 26;15(4):e71763. doi: 10.1002/cam4.71763 (PMC13140982; doi:10.1002/cam4.71763)
Supplement: Supplementary file 1 — Figure S1: Screening of BMR‐DEGs in TCGA‐BC cohort. Figure S2: Validation of BMRG signature in GSE21653 cohort. Figure S3: Nomogram construction in GSE20685 validation set. Figure S4: Immune infiltration and functional analysis. Table S1: Primer sequences used in this study. Table S2: Gene Coefficients and Risk Effects. [file CAM4-15-e71763-s001.docx]

**Supplement Information：**


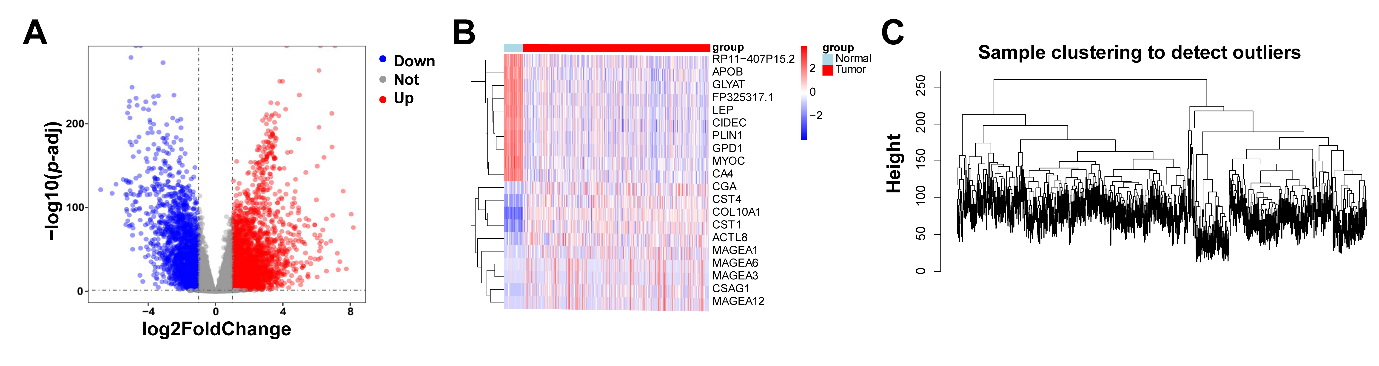


**Supplementary Figure S1. Screening of BMR-DEGs in TCGA-BC cohort.**

(A) Volcano plot of DEGs.

(B) Heatmap of top DEGs.

(C) Sample clustering analysis.


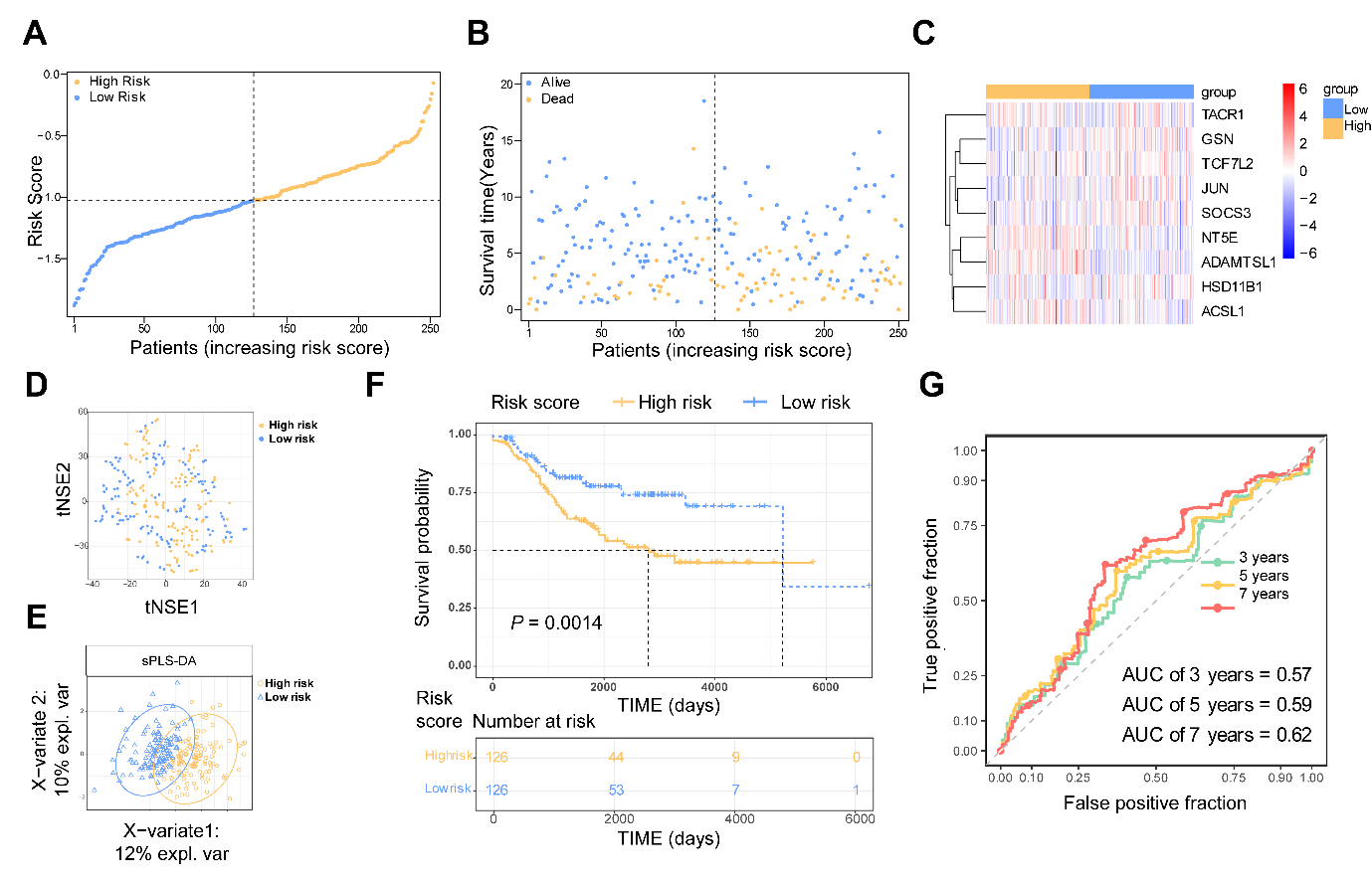


**Supplementary Figure S2. Validation of BMRG signature in GSE21653 cohort.**

(A) Risk score distribution (High-risk, n=126; Low-risk, n=126).

(B) Survival status distribution.

(C) BMRG expression heatmap.

(D) t-NSE analysis plot.

(E) PCA analysis plot.

(F) Kaplan-Meier survival analysis.

(G) Time-dependent ROC analysis.


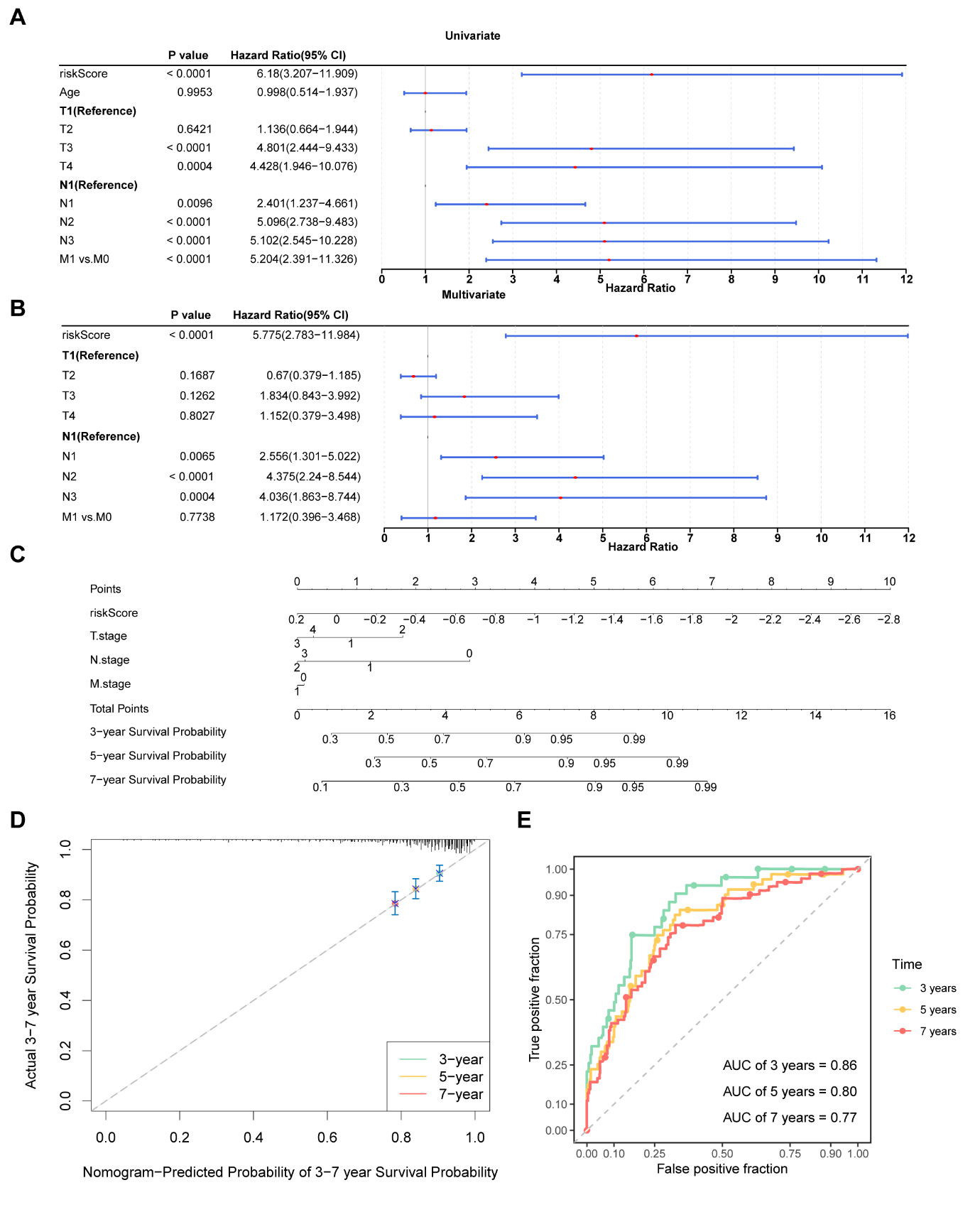


**Supplementary Figure S3. Nomogram construction in GSE20685 validation set.**

(A) Univariate Cox analysis.

(B) Multivariate Cox analysis.

(C) Nomogram diagram.

(D) Calibration curves.

(E) ROC analysis.


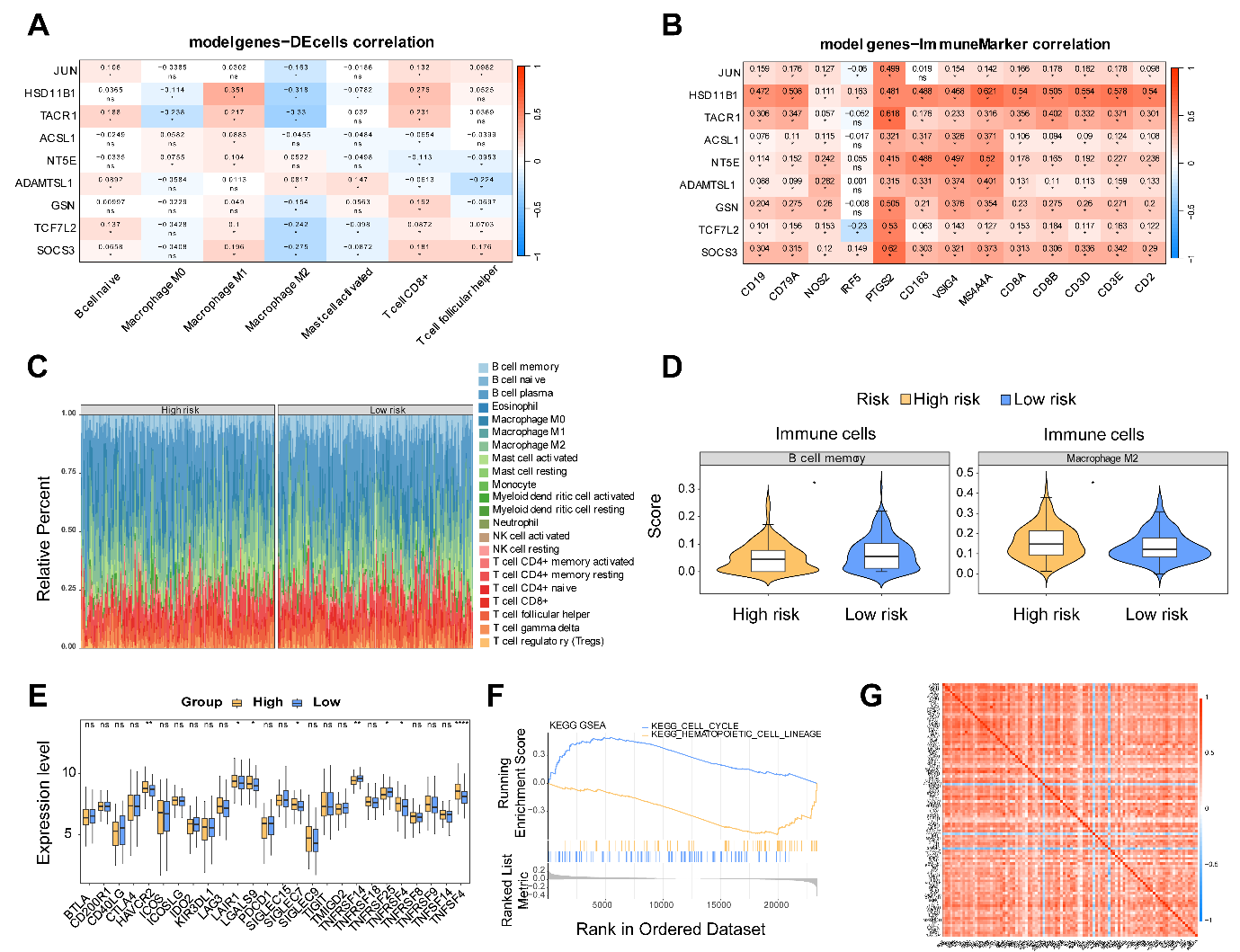


**Supplementary Figure S4. Immune infiltration and functional analysis.**

(A) BMRG-immune cell correlations.

(B) BMRG-immune biomarker correlations.

(C) Immune cell composition.

(D) Differential immune cell infiltration.

(E) Immune checkpoint expression.

(F) GSEA pathway analysis.

(G) BMR-DEGs correlation network. (**p* < 0.05; ***p* < 0.01; ****p* < 0.001; *****p* < 0.0001)

**Supplementary Table S1.** Primer sequences used in this study.

| Gene_name | Sense | Antisense |
| --- | --- | --- |
| JUN | AACAGGTGGCACAGCTTAAAC | CAACTGCTGCGTTAGCATGAG |
| HSD11B1 | CTCAGTTACGTGGTCCTGACT | GAGGAGACGACAACAATGCTT |
| TACR1 | CTAACACCTCGGAACCCAATC | CCACAATGACCGTGTAGGCAG |
| ACSL1 | CCATGAGCTGTTCCGGTATTT | CCGAAGCCCATAAGCGTGTT |
| NT5E | GCCTGGGAGCTTACGATTTTG | TAGTGCCCTGGTACTGGTCG |
| ADAMTSL1 | GGCTTCCTGTGTCTAATGACC | TTAGGTGCTAGTTCAACAACCAG |
| GSN | GGTGTGGCATCAGGATTCAAG | TTTCATACCGATTGCTGTTGGA |
| TCF7L2 | AGAAACGAATCAAAACAGCTCCT | CGGGATTTGTCTCGGAAACTT |
| SOCS3 | CCTGCGCCTCAAGACCTTC | GTCACTGCGCTCCAGTAGAA |
| GAPDH | GTGGACATCCGCAAAGAC | AAAGGGTGTAACGCAACTA |

**Supplementary Table S2:** Gene Coefficients and Risk Effects.

| Gene Symbol | Regression Coefficient | Risk Effect |
| --- | --- | --- |
| JUN | -0.058 | Protective |
| HSD11B1 | -0.159 | Protective |
| TACR1 | -0.033 | Protective |
| ACSL1 | 0.177 | Risk |
| NT5E | 0.129 | Risk |
| ADAMTSL1 | 0.324 | Risk |
| GSN | -0.148 | Protective |
| TCF7L2 | -0.189 | Protective |
| SOCS3 | -0.189 | Protective |

**Raw data in this study.**

1. **The original uncropped images of western blot in this study.**

**Figure 10A.** Original image of ACSL1 and GAPDH in MDA-MB-231 cells.


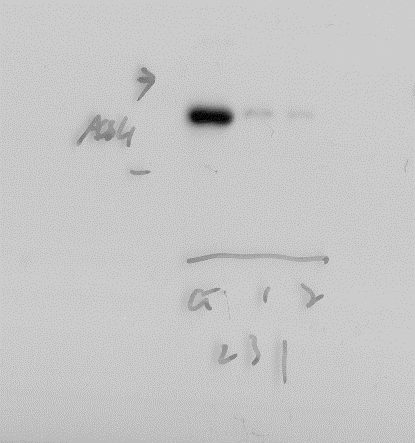


ACSL1


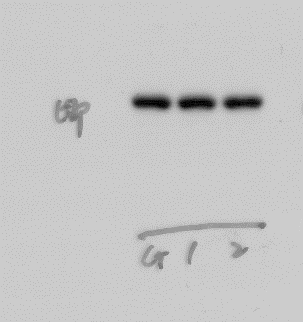


GAPDH

**Figure 10B.** Original image of ACSL1 and GAPDH in MCF7 cells.


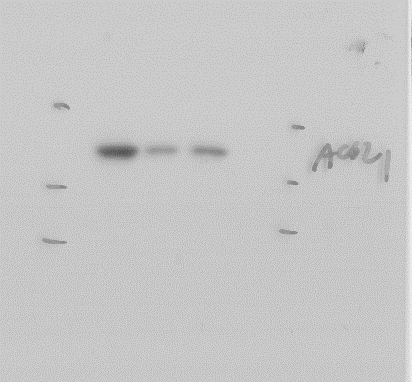


ACSL1


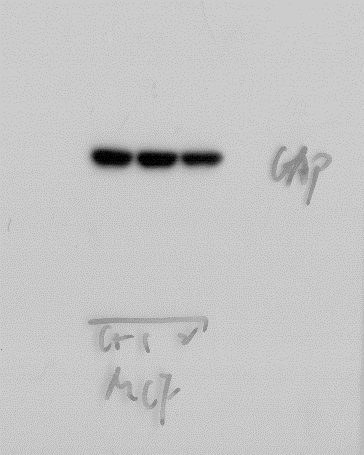


GAPDH

1. **The original microscope images of EdU assay in this study.**

**Figure 10G.**

Original microscope image of MDA-MB-231 cells.

|  | **shGFP** | **sh*ACSL1*** |
| --- | --- | --- |
| EdU | 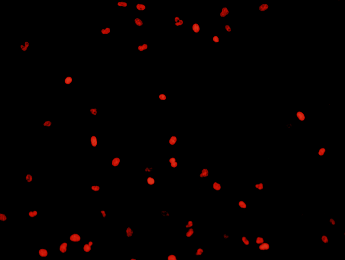 | 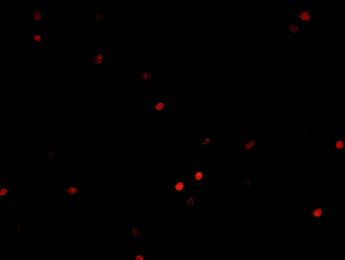 |
| Hochest | 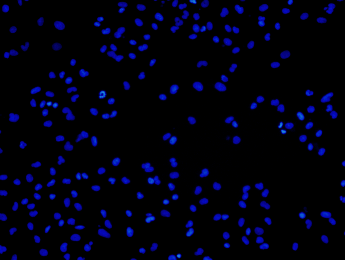 | 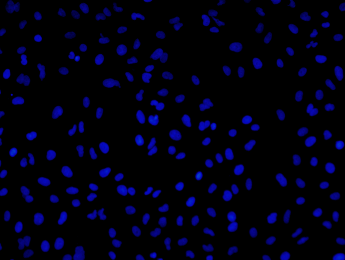 |

Original microscope image of MCF7 cells.

|  | **shGFP** | **sh*ACSL1*** |
| --- | --- | --- |
| EdU | 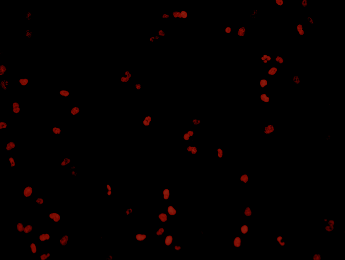 | 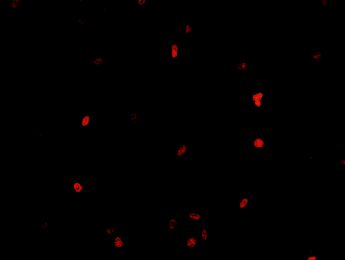 |
| Hochest | 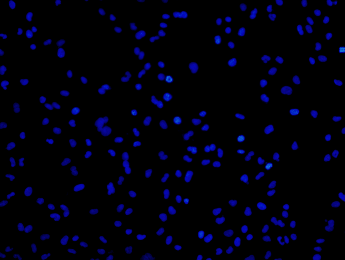 | 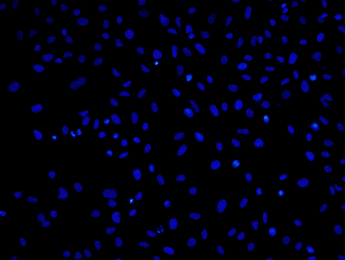 |

1. **The original microscope images of wound healing assay in this study.**

**Figure 10H.**

Original microscope image of wound healing assay in MDA-MB-231 cells.

|  | **shGFP** | **sh*ACSL1_#1*** | **sh*ACSL1_#2*** |
| --- | --- | --- | --- |
| **0h** | **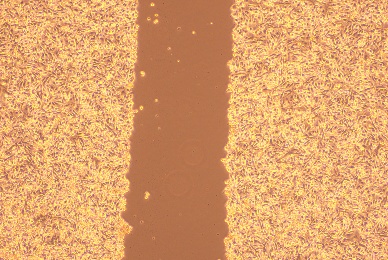** | **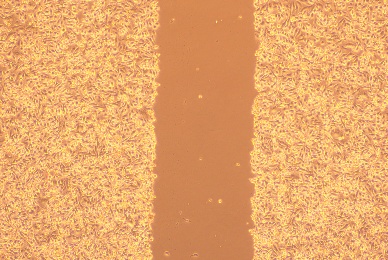** | **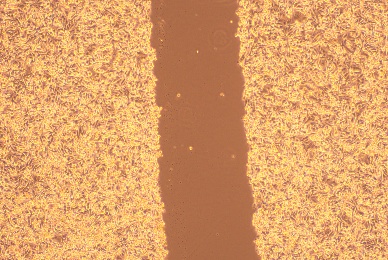** |
| **24h** | **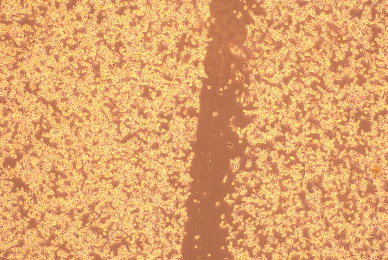** | **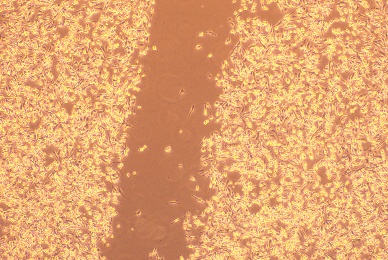** | **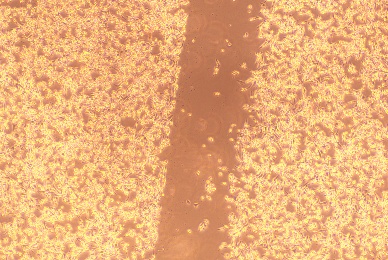** |

Original microscope image of wound healing assay in MCF7 cells.

|  | **shGFP** | **sh*ACSL1_#1*** | **sh*ACSL1_#2*** |
| --- | --- | --- | --- |
| **0h** | **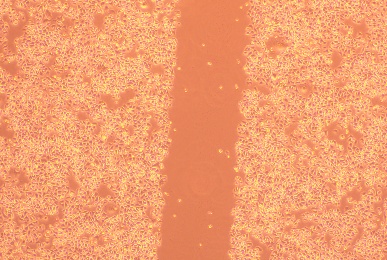** | **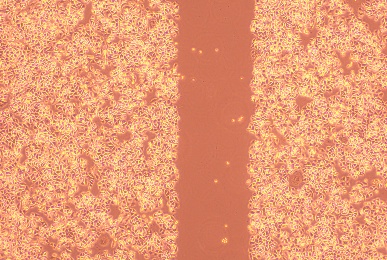** | **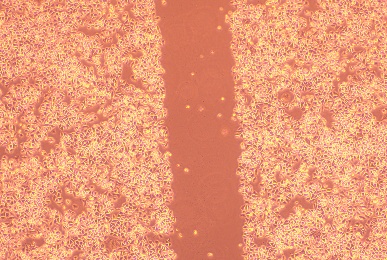** |
| **24h** | **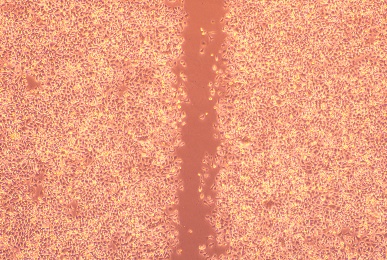** | **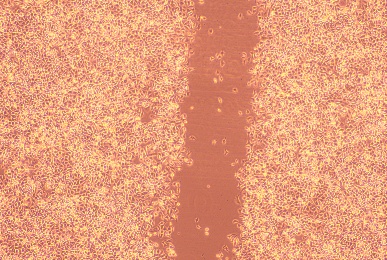** | **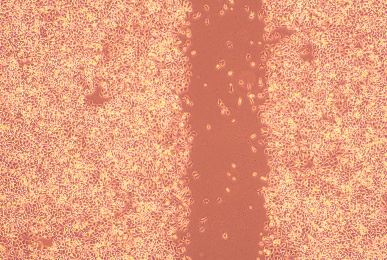** |

1. **The original microscope images of Transwell assay in this study.**

**Figure 10K.** Original microscope image of Transwell assay in MDA-MB-231 cells.

|  | **shGFP** | **sh*ACSL1*** |
| --- | --- | --- |
| **Migration** | 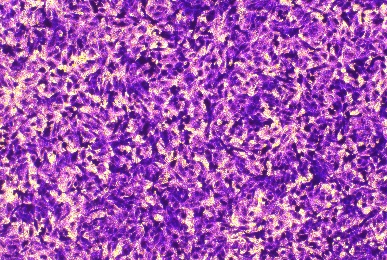 | 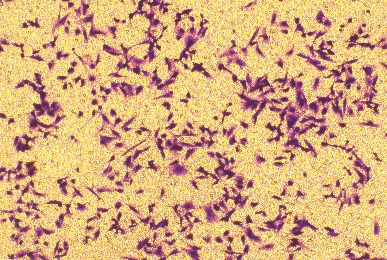 |
| **Invasion** | 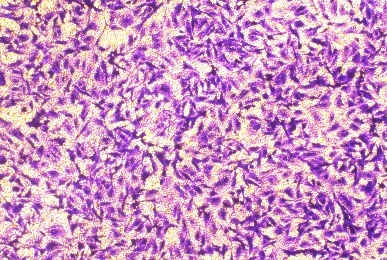 | 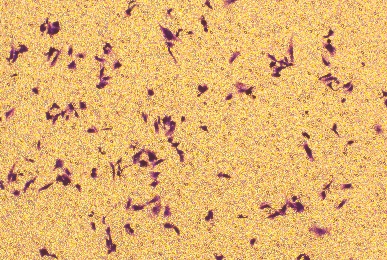 |

Figure 10L. Original microscope image of Transwell assay in MCF7 cells.

|  | **shGFP** | **sh*ACSL1*** |
| --- | --- | --- |
| **Migration** | 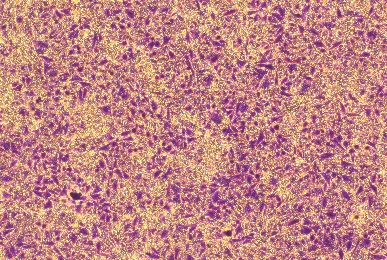 | 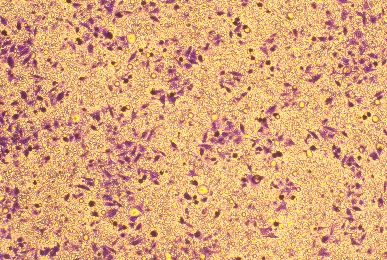 |
| **Invasion** | 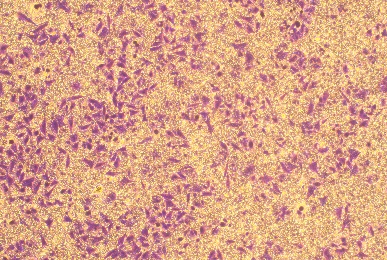 | 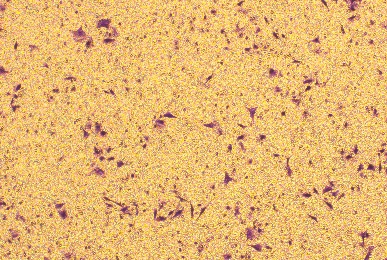 |
